# B cell receptor repertoire analysis in primary Sjogren’s syndrome salivary glands identifies repertoire features associated with clinical activity

**DOI:** 10.1186/s13075-024-03283-z

**Published:** 2024-03-07

**Authors:** Ling Chang, Zihan Zheng, Yiwen Zhou, Kun Liu, Yinong Li, Bing Zhong, Zihua Zhao, Chengshun Chen, Can Qian, Qingshan Ni, Qinghua Zou, Yuzhang Wu, Jingyi Li, Liyun Zou

**Affiliations:** 1 Department of Rheumatology and Immunology, First Affiliated Hospital of Army Medical University, Chongqing, China; 2 Institute of Immunology PLA, Army Medical University, Army Medical University, 30 Gaotanyan Avenue, Shapingba District, Chongqing, 400000 China; 3 Biomedical Analysis Center, Army Medical University, Chongqing, China; 4 Department of Autoimmune Diseases, Chongqing International Institute for Immunology, Chongqing, China

**Keywords:** Sjogren syndrome, pSS, B cell, BCR, IgA2

## Abstract

**Background:**

Primary Sjogren’s syndrome (pSS) is a complex autoimmune disease featuring damage to salivary and lacrimal glands, with the possibility of manifestations across multiple organs. Antibody-producing B cells have long been appreciated to play a significant role in pSS pathogenesis, with a number of autoreactive antibody species having been identified to be elevated in pSS patients. While several studies have attempted to characterize the BCR repertoires of peripheral blood B cells in pSS patients, much remains unknown about the repertoire characteristics of gland-infiltrating B cells.

**Methods:**

Through paired scRNAseq and scBCRseq, we profiled the BCR repertoires of both infiltrating and circulating B cells in a small cohort of patients. We further utilize receptor reconstruction analyses to further investigate repertoire characteristics in a wider cohort of pSS patients previously profiled through RNAseq.

**Results:**

Via integrated BCR and transcriptome analysis of B cell clones, we generate a trajectory progression pattern for infiltrated memory B cells in pSS. We observe significant differences in BCR repertoires between the peripheral blood and labial gland B cells of pSS patients in terms of relative expansion, isotype usage, and BCR clustering. We further observe significant decreases in IgA2 isotype usage among pSS patient labial and parotid gland B cells these analyses relative to controls as well as a positive correlation between kappa/lambda light chain usage and clinical disease activity.

**Conclusions:**

Through BCR repertoire analysis of pSS patient salivary glands, we identify a number of novel repertoire characteristics that may serve as useful indicators of clinical disease and disease activity. By collecting these BCR repertoires into an accessible database, we hope to also enable comparative analysis of patient repertoires in pSS and potentially other autoimmune disorders.

**Supplementary Information:**

The online version contains supplementary material available at 10.1186/s13075-024-03283-z.

## Background

Primary Sjogren’s syndrome (pSS) is a complex autoimmune condition in which damage to salivary and lacrimal glands leads to profound dryness of the mouth and eye [1]. pSS is often accompanied by lethargy and/or other organ manifestations of disease that negatively impacts patient quality of life [2, 3]. A wide range of immune cells have been reported to be involved in the pathogenesis of pSS within salivary glands [4, 5], including T and B cell populations often as part of immune foci and/or tertiary lymphoid structures [6]. Although a number of previous studies have investigated the phenotypes of these lymphocyte populations in terms of their surface marker expression characteristics [7–9] and cytokine secretion profiles [10, 11], much remains unknown regarding the immune receptor repertoires of these populations that dictate their antigen specificity. Indeed, characterization of BCR repertoires in pSS patients has lagged behind efforts to detect autoantibodies and their cognate epitopes. Traditionally, autoantibodies reactive against Ro (SSA) and La (SSB) antigens have been identified to be present in the circulation of a significant proportion of pSS patients and have been associated with disease onset [12, 13]. More recently, other autoantibodies against alpha-fodrin [14], salivary gland protein 1 [15], and muscarinic type 3 receptor [16] have also been reported. However, the lack of sufficient repertoire data of antibody-producing B cells in pSS prevents meaningful exploration of these and other autoreactive clones.

Recently, with the advent of single-cell B cell receptor sequencing (scBCRseq), several studies have reported BCR repertoires of pSS patients. One of these studies collected peripheral blood from six pSS patients for scRNAseq and scBCRseq and identified enrichment in the occurrence of some immunoglobulin heavy chain (IGH) variable-joining gene pairs in pSS patient BCRs [17]. Another study collected the peripheral blood of 24 patients stratified by SSA/SSB autoantibody status and similarly identified differences in the relative usage rates of several IGH variable region genes [18]. These studies have suggested that the peripheral blood BCR repertoires of pSS patients may be significantly altered compared to controls. However, the repertoires of tissue-infiltrating B cells in the salivary glands of pSS patients has yet to be characterized, despite these B cells potentially playing a direct role in disease progression by virtue of their spatiotemporal localization. Furthermore, the extent to which circulating and tissue-infiltrating B cells may share common phenotypes and/or BCR repertoires is also poorly understood.

To help resolve these and other questions, we performed integrated scRNAseq and scBCRseq analysis of paired peripheral blood and labial gland-infiltrating B cells from three pSS patients to generate an initial dataset of paired IgH+IgL BCR repertoires for both tissues. We then supplemented this with additional reconstructed BCR repertoire data from salivary gland bulk RNAseq data, in which variable-joining gene usage, isotype usage, and complementarity determining region 3 (CDR3) sequences could be recovered for individual unpaired heavy or light chains. From these analyses, we identified a number of other repertoire characteristics to vary between pSS patients and controls as well as features correlated with clinical disease activity. We then synthesized the BCR repertoire data into a public resource, which might hopefully act as a stepping stone for further research into the antigen specificity of antibody-producing cells in pSS.

## Materials and methods

### Patient samples

Labial salivary gland biopsies and peripheral blood samples were obtained from a consecutive series of patients with suspected pSS at the Department of Immunology and Rheumatology at the First Affiliated Hospital of Army Medical University, a teaching in Chongqing, China, under approval from the Ethic Committee of the First Affiliated Hospital of Army Medical University (KY2021056). pSS was diagnosed in accordance with criteria from the 2016 ACR/EULAR guidelines [19], and patients presenting with other autoimmune diseases, tumors, or infection were excluded. Three patients fulfilling these criteria were included in our study.

### Library construction and processing

Single-cell BCRseq was performed using a commercially available kit (10X Genomics) using RNA previously isolated from these six samples that had been used for preforming single-cell RNAseq library construction in an earlier work [20]. Cells were matched to their corresponding transcriptomes through their barcodes and processed using the cellranger pipeline (10X Genomics). Cells with incomplete BCR repertoires (missing/incomplete IgH and/or IgL chains) or incomplete complementarity determining region 3 (CDR3) sequences were excluded from analysis. Antibody isotype was recorded based on IgH constant chain gene usage.

### Trajectory analysis

Initial dimension reduction of filtered data was performed using PCA, with samples being integrated together based on patient origin via the harmony package in R [21]. Harmony embeddings were then used as input for UMAP dimension reduction. Trajectory analysis was performed using slingshot [22], a trajectory inference method based on principal curves analysis. To assess the continuity of the inferred trajectories, the scEGRET package in R [23] was used to score trajectories according to gene expression enthalpy. Pathways correlated with progression of the inferred trajectory were identified using the TIPS package in R [24] with slight modification. To identify genes in each of the identified pathways that may be associated with pathway progression, we used LASSO regression to perform feature selection using the glmnet package in R [25]. The gene expression of features with the top coefficients identified by LASSO were visualized against pseudotime progression.

### BCR repertoire reconstruction

In order to recover additional BCR sequences derived from pSS patients and perform comparisons with controls, we downloaded raw .fastq data from a publicly available study reporting bulk RNAseq analyses of labial and parotid gland biopsies taken from pSS patients and controls [26] (GSE173808, accessed through SRA accession SRP318393). Additional .fastq data were downloaded from a biopsy study of minor salivary glands from pSS patients with or without interstitial lung disease (GSE171896) [27]. Adaptive immune receptor repertoire reconstruction was performed using the TRUSTv4 algorithm [28], with BCR sequences with full V, J, C, and CDR3 information being retained for subsequent analysis. Sequences with incomplete information, such as wildcards in the CDR3 region, were filtered out. The remainder were used to help construct a preliminary database of BCR repertoires observed in pSS patients in conjunction with our single-cell RNAseq data. This resource will be made publicly available upon manuscript acceptance in website form (constructed using the shiny package in R) and as a downloadable dataset from GitHub.

### Other analyses and visualizations

BCR diversity was calculated using the abdiv package in R [29]. Shannon diversity of CDR3AA sequences was used as the primary alpha diversity metric. Due to significant variation in the total number of overall BCR counts identified in each sample, we added a correction step of normalizing against the overall BCR count to return the adjusted Shannon diversity values depicted. Repertoire sharing between samples was calculated using the Morisita-Horn similarity index. Most visualizations were depicted using the ggplot2 package in R. Network analysis of BCR CDR3AA sequences, including visualizations and undirected clustering coefficient calculations, were performed using Gephi [30], with the force-directed Fruchterman-Reingold algorithm being used to draw the network graph.

### Statistical analyses

Statistical analyses were performed in R using several packages. All correlational analyses shown were performed using Pearson’s correlation using the base cor function or using the ggpubr package to visualize correlation values directly within plots. Pairwise comparisons were performed where indicated using two-tailed Student’s *t*-test via the stats package in R. *p* values were directly indicated wherever possible; *p* > 0.05 were labeled as not significant (N.S.).

## Results

### Integrated scRNAseq and BCRseq analysis of pSS patients

**Fig. 1:**
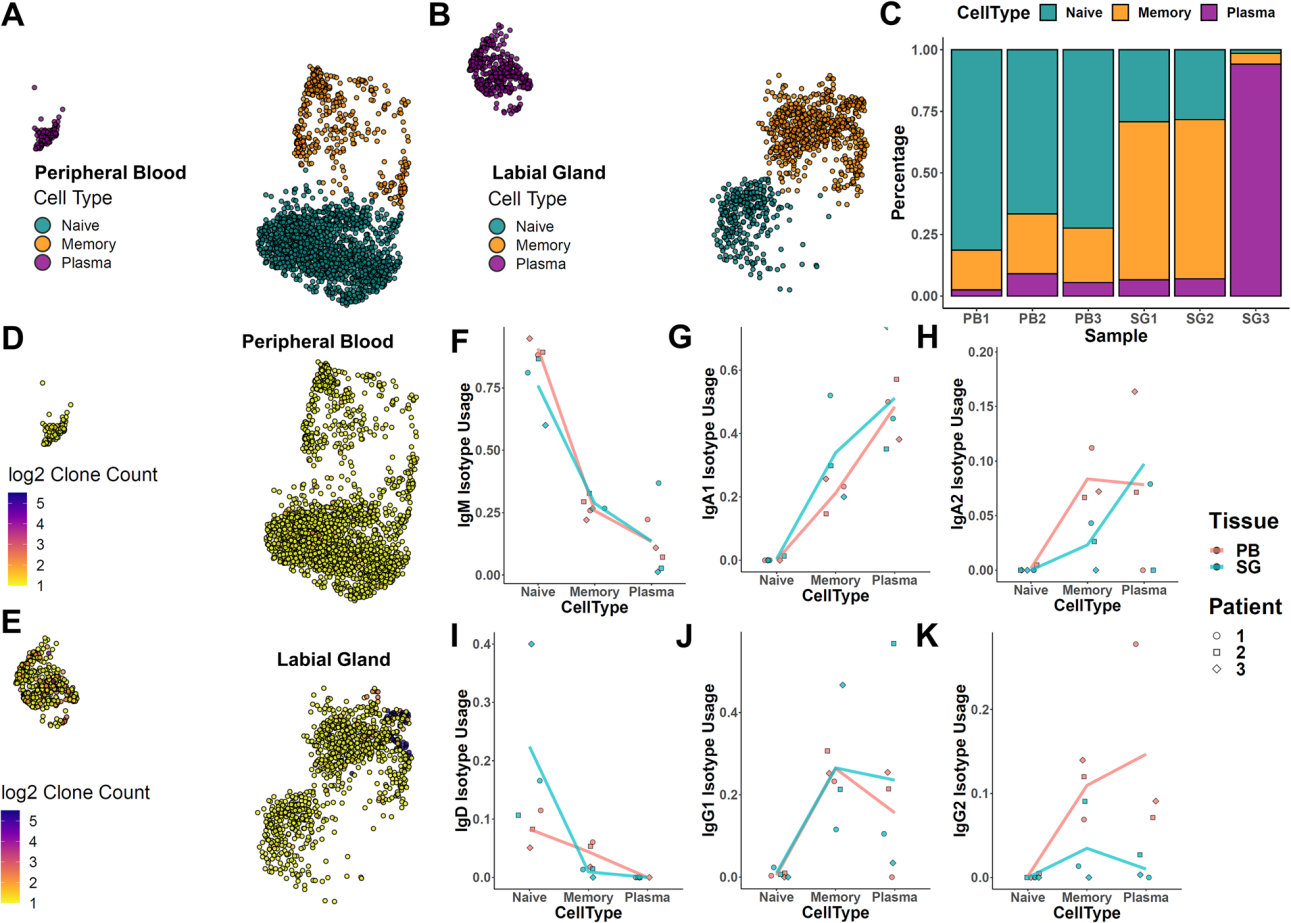
Integrated scRNAseq and BCRseq analysis of pSS patients. Integrated scRNAseq and scBCRseq analysis of B cells taken from paired peripheral blood and labial biopsies of pSS patients (*N* = 3). **A** Projection of peripheral blood B cells of each patient in UMAP space (*N* = 2032 cells). **B** Projection of labial gland B cells of each patient in UMAP space (*N =* 1446 cells). **C** Bar plot of the percentage composition of each sample by cell type. **D**, **E** Visualization of the size of the BCR clone that each cell belongs to, among peripheral blood (**D**) and labial gland (**E**) samples. **F**–**K** Heavy chain isotype usage rates within each B cell population in labial gland and peripheral blood samples, expressed as % of all cells

To gain more insights into the behavior of B cells in pSS, we performed integrated analysis of single B cell transcriptomes from the paired peripheral blood and labial glands of three patients, together with single-cell BCR libraries of these samples. Through dimension reduction and unsupervised clustering, we could classify these B cells into three large subtypes (naïve, memory, and plasma) based on their gene expression characteristics (Fig. S1). From a broad distributional perspective, peripheral blood and labial gland cells showed significant differences space (Fig. 1A, B). Consistent with expectations, naïve cells tended to be underrepresented in labial gland samples, albeit with significant heterogeneity across the three patients (Fig. 1C). By overlaying the BCR sequences of each cell on the UMAP projection, we could observe that only a scattered few peripheral blood cells belonged to the same clonotypes (Fig. 1D), but larger numbers of labial gland memory and plasma populations were expanded (Fig. 1E). While we cannot rule out the possibility that this difference is due to representational variance between the two groups (that these populations in peripheral blood are also expanded, just that their expansion is diluted due to the large numbers of immune cells in circulation), we believe that this result does indicate that expanded antibody-producing cells of the same clonotypes are more highly concentrated in labial glands. Through more detailed examination of the IgH constant chains of these cells, we identified a number of interesting patterns in their isotype usage (Fig. 1F–K). Consistent with expectations, IgM and IgD clones were much less common among memory and plasma cells compared to naïve counterparts in both tissues. However, we observed that labial memory populations had a higher propensity to be IgA1+ than in circulation. In contrast, IgA2+ memory cells were less prevalent in labial memory cells compared to circulation, even though IgA2 usage in plasma cells was similar. At the same time, IgG2+ antibody-producing cells were less prevalent in labial glands by comparison. These results demonstrate that labial gland B cell populations may in fact vary significantly in their repertoires compared with circulating populations.

### Clonal trajectories of memory B cell differentiation in pSS

**Fig. 2:**
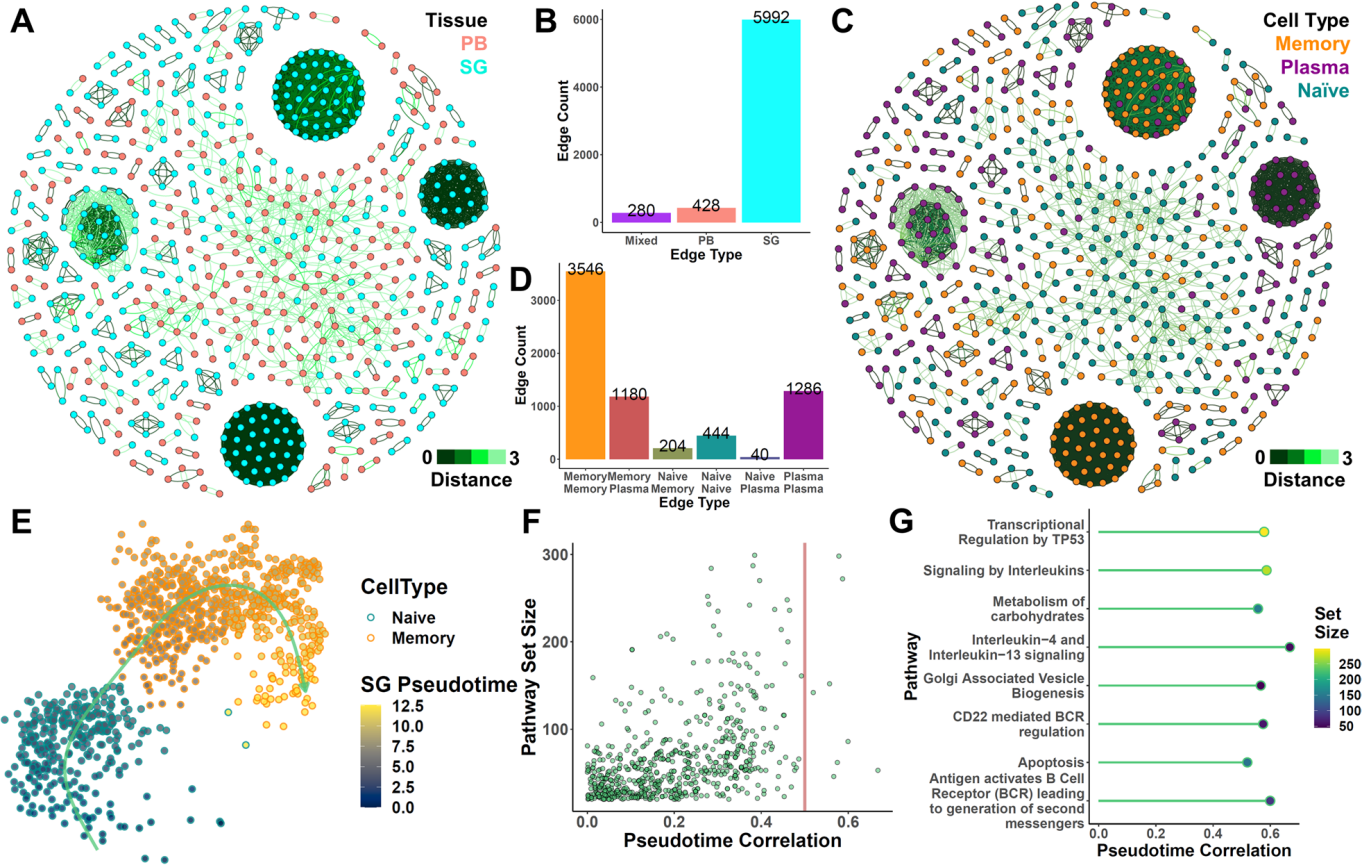
Clonal trajectories of memory B cell differentiation in pSS SGs. **A** Network visualization of all IgH CDR3 amino acid sequences from scBCRseq. Sequences separated by Levenshtein distances < 4 were connected by edges. Each node reflects a single B cell; nodes colored by tissue origin. Four hundred fifteen SG nodes (of 1446 SG cells) and 265 PB nodes (of 2032 PB cells) with at least one connection were retained in the network. **B** Bar plot breakdown of the classification of each edge. Mixed edges connect PB and SG nodes. **C** Network visualization as in **A** with cell types annotated using node color. **D** Bar plot breakdown of each edge according to the connected cell types. **E** UMAP visualization of the inferred trajectory for naïve-to-memory B cell differentiation within labial gland B cells. Arrowed curve indicates direction. **F**–**G** Distribution of the correlation of each Reactome pathway calculated using TIPS with overall pseudotime progression (**F**). Eight pathways with correlation > 0.5 are shown in **G**

After observing significant differences in the BCR repertoire characteristics of labial B cell populations, we then sought to characterize the clonal characteristics and differentiation trajectory of these populations. We first performed sequence clustering of the IgH repertoires for each cell identified. Network analysis of the linked nodes demonstrated that SG cells were more likely to be associated into BCR clusters, while PB clusters were relatively uncommon, and clusters containing sequences found in both PB and SG were even rarer (Fig. 2A, B). This result suggests that an examination only considering circulating or labial B cell repertoires may not necessarily capture a representative reflection of the other. On a more general level, however, some clusters featured sequences shared across patients (~6% of all edges, Fig. S2), suggesting that a portion of the repertoires of these patients may be similar.

At the same time, when we examined repertoire sharing across cell types, we found that memory cell connections were the dominant edge type, while sizeable portions of memory-plasma (albeit predominantly limited to a single, large clonotype cluster) and naïve-memory (represented by multiple clusters) edges could also be detected, while naïve-plasma connections were rare (Fig. 2C, D). These patterns are consistent with pre-existing expectations that naïve clones can differentiate into memory cells and that memory cells might further differentiate into plasma cells. To further confirm this observation, we then repeated this analysis using paired heavy and light chains. In this strict clustering analysis, we identified fewer potentially connected sequences, with very little overlap across patients under the most stringent criteria (Fig. S3A) or across isotype classes (Fig. S3B). However, we once again observed a number of clones to fall in shared clusters spanning naïve and memory B cell states and a smaller number spanning memory and plasma phenotypes (Fig. S3C). As such, these results further support our hypothesis that the three B cell populations we observed may be clonally linked, particularly in the salivary glands.

To further describe the progression patterns of B cells in pSS patients, we then utilized trajectory analysis using the scRNAseq profiles of these cells. We first attempted to perform an integrated trajectory encompassing all three B cell types (Fig. S4A-B). However, continuity assessment of this trajectory using scEGRET demonstrated that plasma cells were too widely distinct from naïve and memory B cells in this context, such that an accurate and “complete” trajectory could not be meaningfully inferred from transcriptome data alone using currently available computational methods (Fig. 4C, D). This is perhaps unsurprising given that plasma cell differentiation is a complex process which may potentially require a plasmablast intermediary with its own unique characteristics or might otherwise involve a very rapid change in transcriptome profile that is difficult to capture in unbiased sequencing. In the interest of performing a more robust analysis, we thus restricted our trajectory to only consider naïve and memory populations, which we found to be connected via multiple clonotypes (Fig. 2E). Pathway progression analysis of this trajectory using TIPS identified 8 known pathways to have significant associations with memory cell differentiation (Fig. 2F). Interestingly, these include both well-characterized contributors, such as IL4-IL13 signaling and antigen-driven BCR activation as well as other processes such as Golgi-associated vesicle biogenesis and carbohydrate metabolism (Fig. 2G). From LASSO-based feature selection, we were able to identify a number of genes in each of these pathways with expression patterns that were associated with pseudotime progression (Fig. S5). In the IL4-IL13 signaling gene list, we could observe decreased *S1PR1* and increased *IRF4* (Fig. S6A). In the BCR antigen activation pathway, we observed decreased in *IGHM* and increased *IGKC*, consistent with B cell class-switch recombination following activation (Fig. S6B). From the Golgi-associated vesicle list, we observed an interesting increase in *FTL* and *TFRC* expression, suggesting a potential role for iron transport (Fig. S6C). Inspection of the carbohydrate metabolism processes showed involvement of several enzymes involved in sugar processing, such as *GAPDH*, *ENO1*, and *GNPDA1* (Fig. S6D). Collectively, these results generally support the notion that the pathways we identified to be involved in trajectory progression of labial gland B cell memory differentiation were in fact correctly identified based on involvement of relevant factors. Taken together with our BCR repertoire analyses, these results thus give some preliminary support to the hypothesis that some naïve B cells in the labial gland might undergo local differentiation into memory cells.

### Database of observed BCRs in pSS

**Fig. 3:**
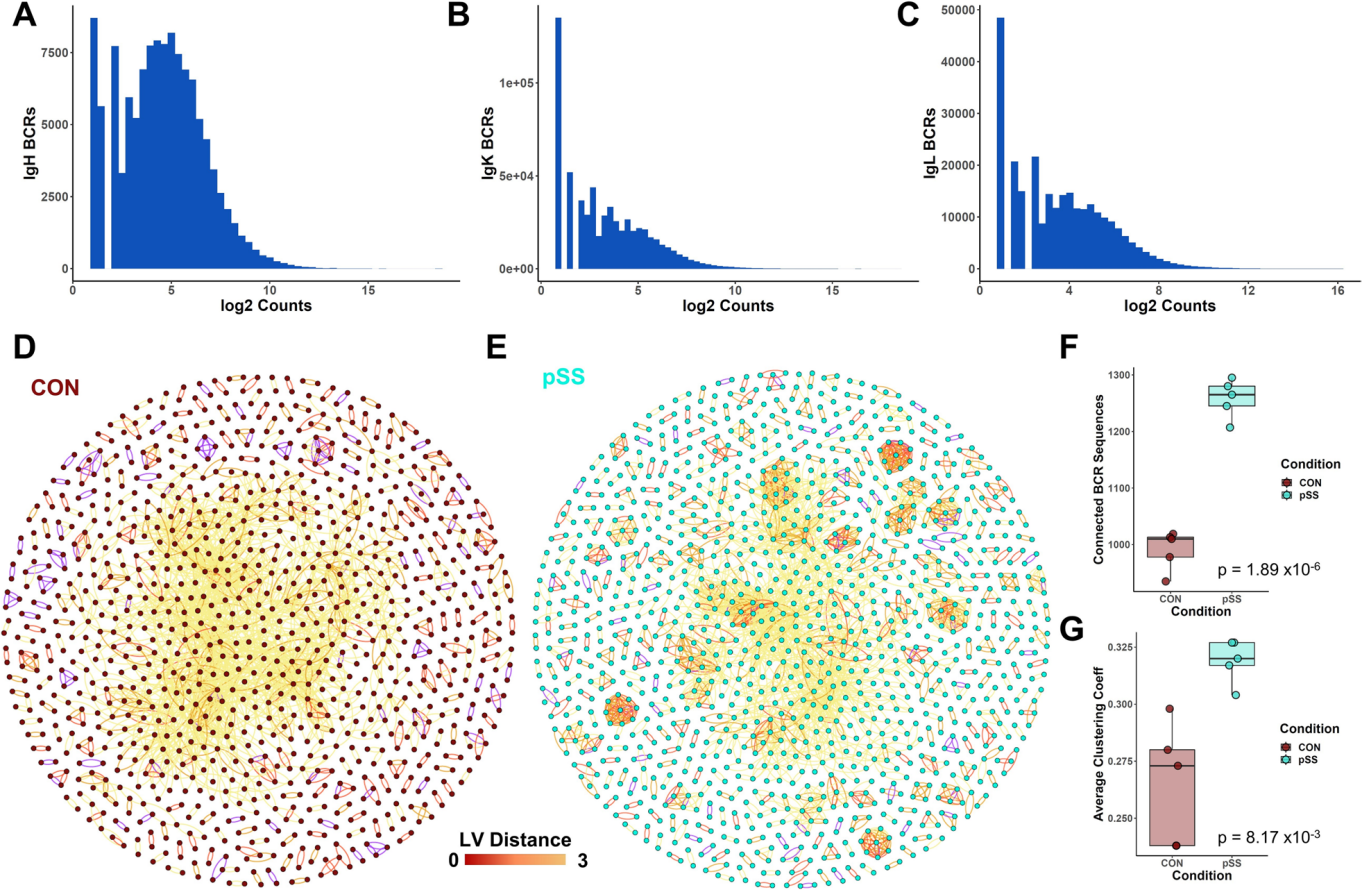
Building a database of observed BCRs in pSS. **A**–**C** Histogram mapping the count distribution of each observed gland-infiltrating B cell BCR, segregated by chain. **D** Network visualization of clustering for 5000 randomly downsampled CDR3 AA sequences taken from controls. Nodes with LV distance < 4 are connected by edges. **E** Clustering of 5000 randomly downsampled sequences, as in **D**, of sequences from pSS patients. **F** Summary of connected node count in the control and pSS groups (5 random downsamples). Student’s two-tailed *t*-test *p* = 1.89 × 10^−6^. **G** Summary of the average clustering coefficient of each network, calculated in Gephi. Student’s two-tailed *t*-test *p* = 8.17 × 10^−3^

In order to better understand the extent to which the BCR-associated inferences we drew above may be relevant to clinical disease activity, we then attempted to compare our analysis inferences with external datasets. Given the lack of publicly available BCR repertoire data in pSS however, we were unable to find a reference for direct comparison. Instead, we were only able to find a previous study that performed RNAseq of pSS patient and control salivary gland biopsies. As such, we next utilized immune receptor repertoire reconstruction to recover BCR sequences from this source to build an initial dataset of the observed BCR repertoire of pSS patients. Through this analysis, we recovered > 1 × 10^5^ high confidence IgH chain sequences from 114 samples, with most of these chains being supported by multiple reads (Fig. 3A). And while the nature of repertoire reconstruction did not permit us to identify heavy-light chain pairs as in scBCRseq, we also recovered sizeable numbers of high confidence IgL and IgK chain sequences (Fig. 3B, C). As a preliminary analysis, we randomly selected 5000 IgH BCR CDR3 sequences each from control and pSS patients in our reconstructed dataset. By clustering these CDR3 sequences according to their string distance, we could observe in the resulting network that the sequences taken from pSS patients showed an apparent tendency to more readily form clusters (Fig. 3D, E). This observation could be supported by statistical analysis of the number of connected BCR sequences (Fig. 3F) as well as average network clustering coefficient (Fig. 3G). Taken together, these results form a preliminary BCR repertoire database for performing comparisons in pSS patients and hint at the possibility that BCR sequences in pSS patients may be more similar in terms of sequence than in healthy control. To make this repertoire resource accessible, we have compiled these recovered sequences into a publicly available database.

### pSS patient BCR characteristics correlate with clinical disease activity

**Fig. 4:**
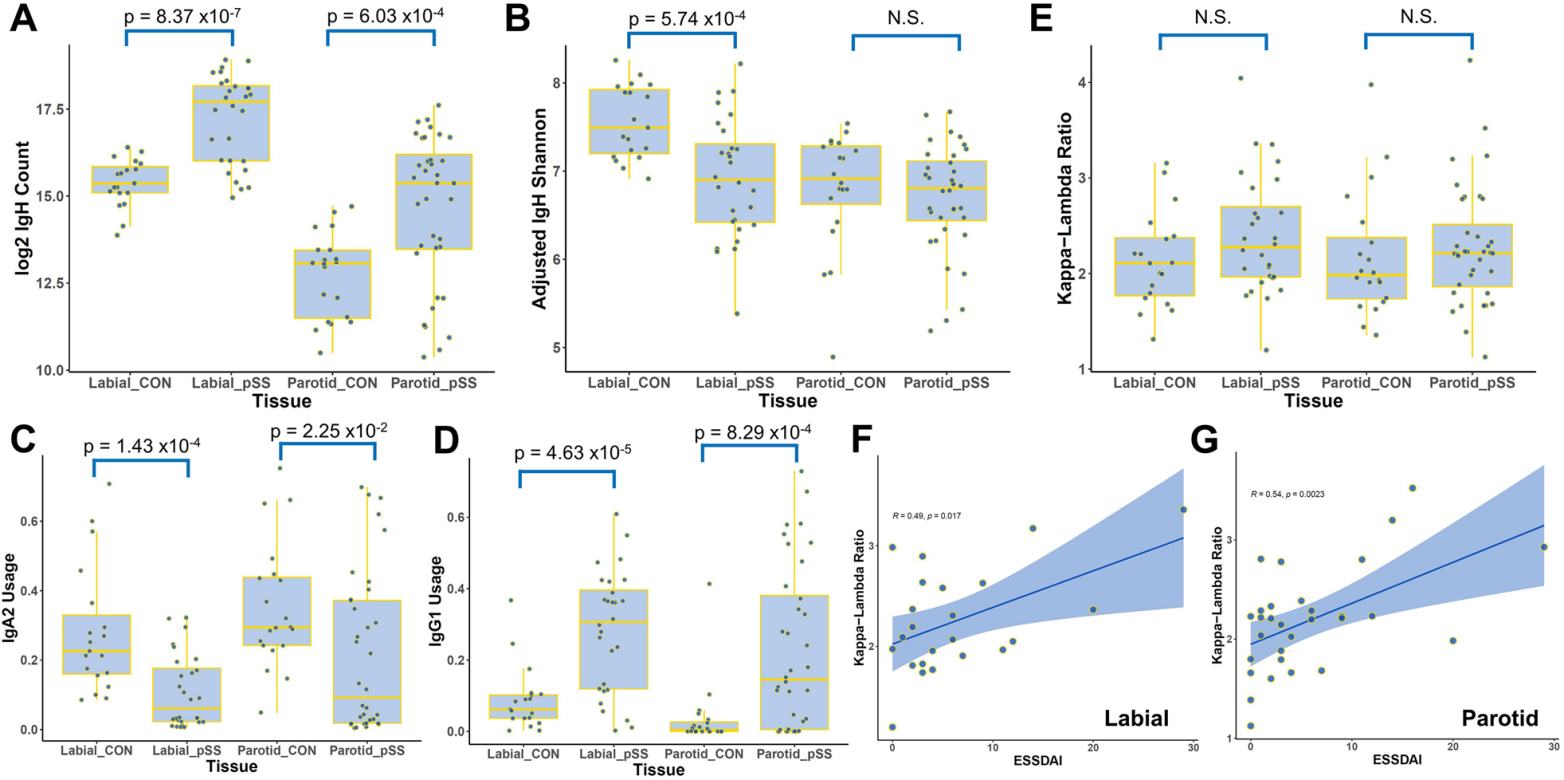
pSS patient BCR *κ*-*λ* usage ratios correlate with clinical disease activity. **A** Boxplot of the total counts of recovered IgH chains in each sample from the labial and parotid glands of pSS patients and controls. *p* values of pairwise two-tailed Student’s *t*-test depicted. **B** Boxplot of the adjusted Shannon entropy of IgH chain CDR3 amino acid sequences in each sample. To normalize for differences in total count, raw Shannon entropy was divided by a scale factor for total IgH count in each sample. **C**, **D** Boxplot of the IgA2 isotype usage rate (**C**) and IgG1 isotype usage rate (**D**) as a proportion of total IgH counts in each sample. **E** Boxplot of light chain *κ*-*λ* usage ratio in each sample shows no significant variation. **F**, **G** Kappa-lambda ratio of labial gland (**F**) and parotid gland (**G**) BCRs is positively correlated with ESSDAI disease activity metric. Correlation coefficients and *p*-values calculated using Pearson’s R

Beyond the preliminary sequence-based analyses we performed above, we further analyzed the correspondence between more general repertoire metrics and clinical disease activity using this dataset. Consistent with expectations, we observed that both labial and parotid glands derived from pSS patients had higher counts of recoverable IgH chains compared to controls (Fig. 4A). Labial glands of these pSS patients also showed reduced Shannon diversity compared to control, suggestive of increased clonal expansion (Fig. 4B). However, we only observed suggestive, and not statistically significant, correlations between these metrics and clinical disease activity of these patients as measured through the EULAR Sjogren’s syndrome disease activity index (ESSDAI) (Fig. S7, S8). These results suggest that these general measures of B cell presence may only be suitable for distinguishing pSS patients from controls and may not necessarily robustly reflect dynamic changes in clinical disease activity.

We then sought to evaluate patterns of isotype usage in this dataset. Interestingly, overall IgA2 clones were significantly underrepresented in the labial and parotid glands of pSS patients compared to controls (Fig. 4C). Taken together with our scBCRseq observation that memory IgA2 clones were less common in affected glands compared with circulation, this result suggests that IgA2 antibodies might be less likely to be associated with pSS clinical activity. IgA1 usage did not show a significant decrease (Fig. S9A). At the same time, we observed that labial gland IgM usage was actually slightly higher in pSS patients compared with control, while there was little to no difference in IgD (Fig. S9B-C). In contrast, usage rates of IgG1, but not the other IgG isotypes, was significantly increased in pSS patients (Fig. 4D, S9D-F). Collectively, these results support our inferences from scBCRseq that isotype usages may vary significantly in pSS patients.

At the same time, the *κ*-*λ* light chain usage ratio was not significantly varied in pSS patients relative to control (Fig. 4E). However, *κ*-*λ* ratio did show a significant positive correlation with clinical disease severity in both labial and parotid biopsies (Fig. 4F, G).

Our calculation of *κ*-*λ* ratio here is distinct from the commonly cited clinical assay (which instead assesses the concentration of free *κ* and *λ* light chains in circulation). However, this result suggests that light chain recombination and relative usage patterns might also be explored in association with pSS disease activity. These results further support our hypothesis that BCR repertoires may also be substantially skewed in the affected salivary glands of pSS patients, and highlight *κ*-*λ* ratio as a possible marker of disease activity.

Since *κ*-*λ* ratio in both labial and parotid glands was found to be positively associated with ESSDAI, we next explored the clonal sharing between paired labial and parotid glands of the same patients. To our surprise, BCR repertoire similarity was not higher in pSS patients compared with control; in fact, IgK chain similarity was significantly decreased (Fig. S10A-C). However, we more generally observed that the overall BCR diversity for heavy and light chain CDR3 regions were more similar in pSS patients compared with controls (Fig. S10D-F). These results thus suggest that the expanded clones in parotid and labial glands of pSS patients may not necessarily show strong clonal overlap and raise the possibility that different antigens may be involved at the two tissue sites.

## Discussion

IgA antibodies have long been understood to play a dominant role in host protection against pathogens at mucosal membranes and in circulation. In humans, two forms of IgA- IgA1 and IgA2 are commonly found, with both capable of forming soluble dimeric complexes in the secretory form and single monomers in the serum form [31]. Dedicated Fcα receptors expressed by macrophages, neutrophils, and other innate immune cells can recognize serum, but not secretory, IgA, to trigger their activation [32]. Interestingly, a recent study has demonstrated that serum IgA2 may be more potent at stimulating macrophage and neutrophil activity compared to serum IgA1 by virtue of having fewer O-glycosylation sites [33]. In our current study, we identify that IgA2 is instead less commonly used among memory B cells in the labial glands as compared with peripheral blood in pSS patients, while IgA1 usage is increased. We also observe that IgA2 usage is lower in salivary glands of pSS patients compared with controls, with differences in IgA1 usage being insignificant. These results thus suggest that IgA2 may actually be a protective marker in the context of pSS, at least on a transcriptional level. However, additional studies on the monomeric/dimeric status and glycosylation profiles of IgA1 and IgA2 antibodies in pSS patients is necessary to draw clear insights into this question. After all, aberrant glycosylation of IgA1 is also known to contribute to the formation of persistent immune complexes and inflammation, such as in the context of IgA nephropathy. It may also be possible then that an alternative glycosylation profile may also cause IgA2 antibodies to adopt a regulatory function. Such a posture might also explain the observations in other reports that Fcα receptor signaling can instead inhibit innate immune cell responses [34, 35]. Resolution of this question may help to significantly expand our understanding of the functional roles of each antibody isotype.

Besides our observations of variation in heavy chain isotype usage, we also observed a correlation between *κ* and *λ* light chain usage and disease activity in pSS patients. While our assessment of *κ*-*λ* ratio here is on a transcriptional level, these findings are roughly consistent with the pervasive observations that higher *κ*-*λ* ratios correspond to increased inflammatory activity across a wide range of human pathologies [36, 37]. However, despite the extensive use of *κ*-*λ* ratios as a biomarker in these reports, the molecular mechanisms that contribute to increases in *κ*-*λ* ratios are very poorly understood. Unlike in heavy chain class switching, where recombination is relatively well characterized, isotypic selection in light chains is poorly understood, with some studies suggesting that *κ* chains have higher recombination likelihood compared to *λ* chains [38, 39]. The functional consequences of *κ* versus *λ* chain usage are also unclear, and some studies have even suggested that both chains could be present in the same B cell [40]. However, the observation that *κ* chain usage is increased in some contexts suggests that light chain isotype usage is likely subject to some kind of mechanistic regulation in at least a portion of B cells. After all, if *κ* and *λ* chains could be completely randomly selected, clonal selection would be unlikely to exclusively favor *κ* clones to expand over *λ* clones, since the ratio between them is typically around 2:1 under physiological conditions. Further investigations are warranted to this question.

Beyond our observations of BCR repertoire differences in pSS patients, we have also assembled in this manuscript a preliminary BCR database resource for pSS patients from our scBCRseq and reconstructed repertoire data. From our current dataset, we already have observed significant differences in BCR repertoires between the peripheral blood and labial glands of pSS patients, including a trend towards higher clonal similarity in pSS patient labial gland samples compared to both peripheral blood and control labial glands. While we cannot confirm that these labial gland B cells are necessarily autoreactive and/or pathogenic, we hypothesize that it is more likely that pathogenic B cells will have accumulated in the affected glands and may directly secrete a significant portion of the autoantibodies deposited in the glands. By continuously accumulating BCR repertoire data of pSS patients, it may become possible to characterize the degree to which pSS patients possess public antibody sequences and identify common antigens that patients are responsive to in the future. This latter potential also speaks to a significant limitation of our current work, as we are currently unable to draw any clear conclusions regarding the antigen specificity of the BCR repertoires we have reported here. However, continued advances in antigen screening and sequencing technologies may make it possible to establish the actual antigen specificity of each BCR over time [41]. Future efforts in this vein may help to identify common antigens in pSS patients that can then serve as therapeutic targets, and lead to the development of effective therapeutics for pSS.

## Conclusion

Through integrated BCR and transcriptome sequencing, we report here the first single-cell profiles of B cells in the labial glands of pSS patients. These gland-infiltrating B cells show marked differences compared to peripheral blood populations in terms of expansion, isotype usage, and BCR clustering characteristics. We further validate a number of these findings through comparisons of labial and parotid gland BCR repertoires of pSS patients and controls, as facilitated by repertoire reconstruction analysis. Collectively, these findings and the combined resource formed therein may be useful for identifying repertoire-based biomarkers of clinical disease and disease activity in pSS patients. Cross-comparisons with data generated in other autoimmune diseases and antigen validation studies may help elucidate patterns of autoreactive B cell behavior.

### Supplementary Information

**Additional file 1:** **Figure S1.** Gene expression differences between B cell populations in labial and peripheral blood samples. A) Heatmap of the top 50 differentially expressed genes marking each of the three cell types we annotated. B-D) Top differentially expressed genes among naïve B cells (B), memory B cells (C), and plasma cells (D) between peripheral blood and SG samples. DEGs calculated using Wilcoxon test, with adjusted *p* values < 0.05. Genes expressed in fewer than 20% of cells of at least one group were excluded. **Figure S2.** BCR clustering based on IgH CDR3 AA sequence. A) Network visualization as in Fig. 2A, annotated according to patient origin. B) A) Network visualization as in Fig. 2A, annotated according to heavy chain isotype usage. **Figure S3.** BCR clustering and associated isotypes using strict clustering of paired IgH and IgL/IgK chains. Network visualization of BCR repertoire sequences clustering by paired CDR3 amino acid sequence; sequences with Levenshtein distance between 1 and 3 are connected by edges. A) Nodes annotated according to patient origin. B) Nodes colored according to heavy chain isotype. C) Nodes colored according to cell type. **Figure S4.** Plasma cell differentiation in pSS SGs does not form a continuous trajectory. A) Trajectory analysis of all labial gland B cells using slingshot infers a lineage connecting naïve, memory, and plasma states. B) Stream plot of the distribution of each cell type in each bin of the pseudotime trajectory. C) Pyramid heatmap of the number of differentially expressed genes from bin-to-bin along the pseudotime trajectory. A large number of DEGs can be seen in the step that would cross from memory cell to plasma cell, causing an inferred break in the trajectory. D) Relative step change across each pseudotime step shows a break in the trajectory corresponding to the plasma cell crossover point. Since currently available trajectory inference algorithms implicitly presume continuity, a lack of continuity in a real-world dataset will likely lead to the trajectory inference being error-prone and oftentimes nonsensical. **Figure S5.** LASSO selection of key pathway features. To identify genes associated with pseudotime progression from each of the four pathways noted, we used LASSO for feature selection based on the scaled gene expression of each feature. A-D) Mean-squared error plots for each pathway. E-H) Correlation coefficients of each model with respect to pseudotime progression. While the model is unsurprisingly noisy given the technical dropout in single cells, correlation values are at least better than 0.6 for these four pathways. **Figure S6.** Gene expression change with respect to pseudotime in selected pathways. A-D) Scaled gene expression patterns over the course of trajectory progression of LASSO-selected features identified in each of the four pathways selected. **Figure S7.** pSS patient glandular BCR counts is only weakly associated with disease activity metrics. A) Correlation between total log-normalized IgH read counts and ESSDAI in labial (top) and parotid (bottom) samples. Non-pSS samples were excluded. Correlations given as Pearson’s R. B) Correlation as in (A) of log-normalized IgK light chain counts and ESSDAI. C) Correlation as in (A) of log-normalized IgL light chain counts and ESSDAI. **Figure S8.** pSS patient BCR diversity is not clearly associated with disease activity metrics. A) Correlation between count-adjusted Shannon entropy of IgH CDR3 AA sequences and ESSDAI in labial (top) and parotid (bottom) samples. Non-pSS samples were excluded. Correlations given as Pearson’s R. B) Correlation as in (A) of count-adjusted Shannon entropy of IgK light chain CDR3 AA and ESSDAI. C) Correlation as in (A) of count-adjusted Shannon entropy of IgL light chain CDR3 AA and ESSDAI. **Figure S9.** BCR isotype usage rates in salivary glands. Bar plots of isotype usage rates for 6 other Ig isotypes compared between pSS patients and controls for both labial and parotid glands (see also Fig. 4C, D). One other isotype, IgE, went essentially undetected and was excluded from analysis. **Figure S10.** BCR repertoire sharing between parotid and labial glands. A-C) Bar plot of repertoire similarity of IgH (A), IgK (B), and IgL (C) CDR3 AA sequences between labial and parotid glands of pSS patients and controls, calculated using Morisita-Horn similarity (MHS). Only IgK shows a significant difference between the two groups, with pSS samples being more dissimilar compared with controls. D-F) Differences in adjusted Shannon entropy between labial and parotid gland samples of pSS patients and controls for IgH (D), IgK (E), and IgL (F) CDR3 AA sequences. p values calculated using Student’s two-tailed t-test.

## Data Availability

Sequencing data for pSS patients in this study are available through the Genome Sequence Archive under accession HRA006656. Reconstructed immune repertoire sequencing data are accessible online through [http://118.24.236.198:3838/pss/]. Any other requests for data will by met by the corresponding author L.Z.

## Abbreviations

pSS: Primary Sjogren’s syndrome
BCR: B cell receptor
scBCRseq: Single-cell B cell receptor sequencing
scRNAseq: Single-cell mRNA sequencing
IgH: Immunoglobulin heavy chain
IgL: Immunoglobulin lambda light chain
IgK: Immunoglobulin kappa light chain
CDR3: Complementarity determining region 3
ESSDAI: EULAR Sjogren’s Syndrome disease activity index
UMAP: Uniform manifold approximation and projection
LASSO: Least absolute shrinkage and selection operator
AA: Amino acid
