# Supplementary material for: B cell receptor repertoire analysis in primary Sjogren’s syndrome salivary glands identifies repertoire features associated with clinical activity
Source: Arthritis Res Ther. 2024 Mar 7;26:62. doi: 10.1186/s13075-024-03283-z (PMC10918881; doi:10.1186/s13075-024-03283-z)

Figure S1– Gene expression differences between B cell populations in labial and peripheral blood samples

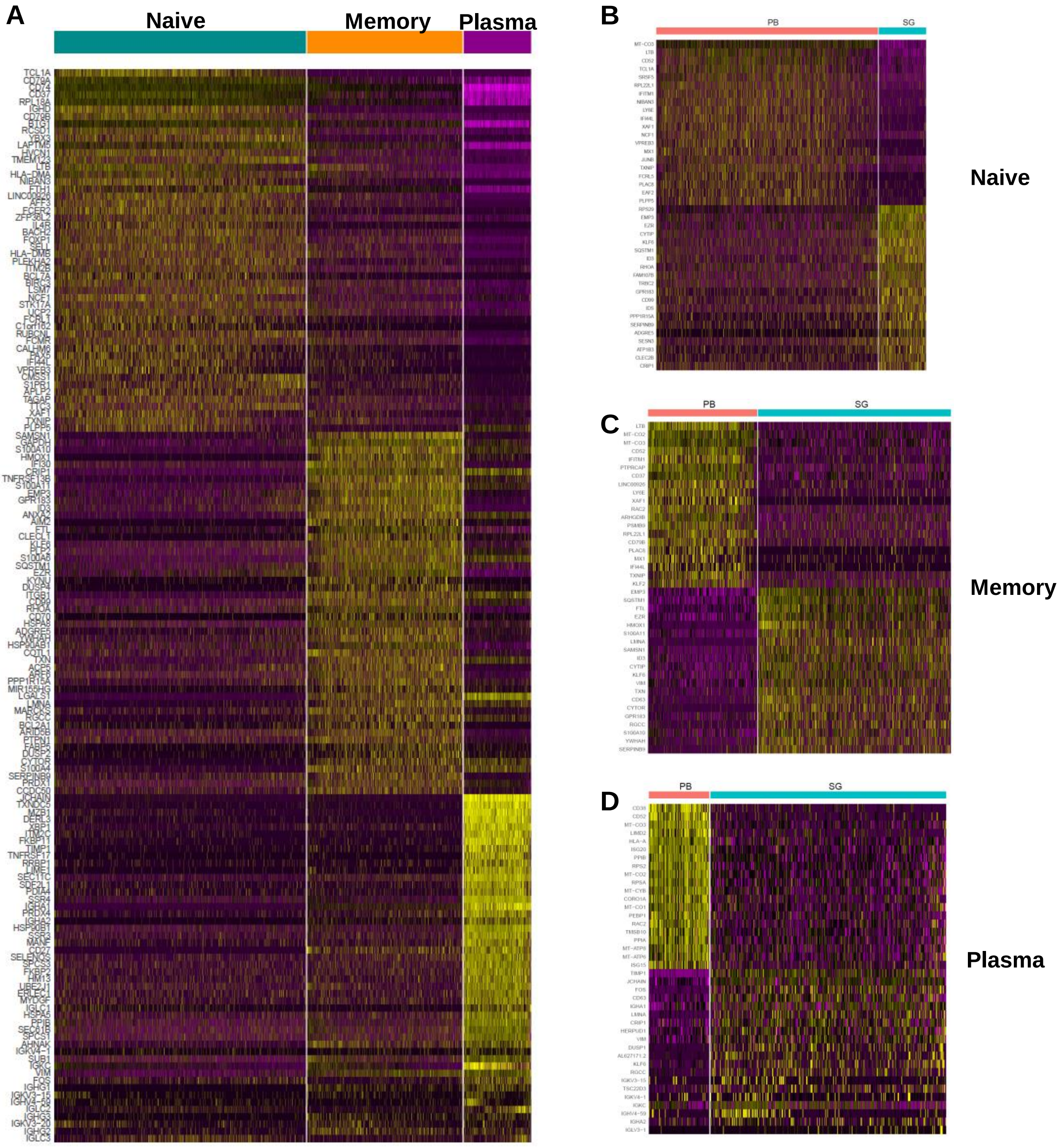

Figure S2– BCR clustering based on IgH CDR3 AA sequence

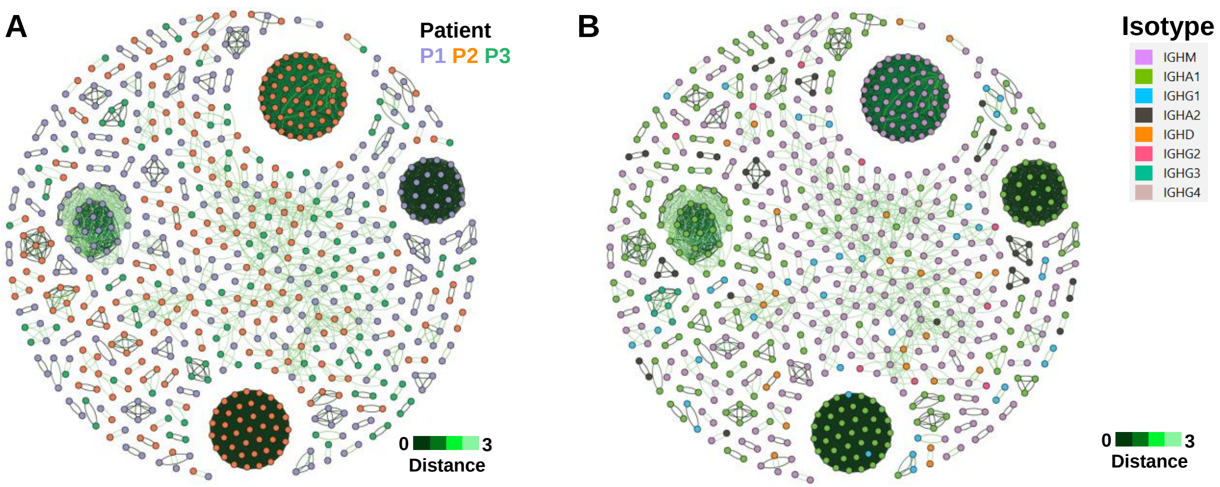

Figure S3– BCR clustering and associated isotypes using strict clustering of paired IgH and IgL/IgK chains

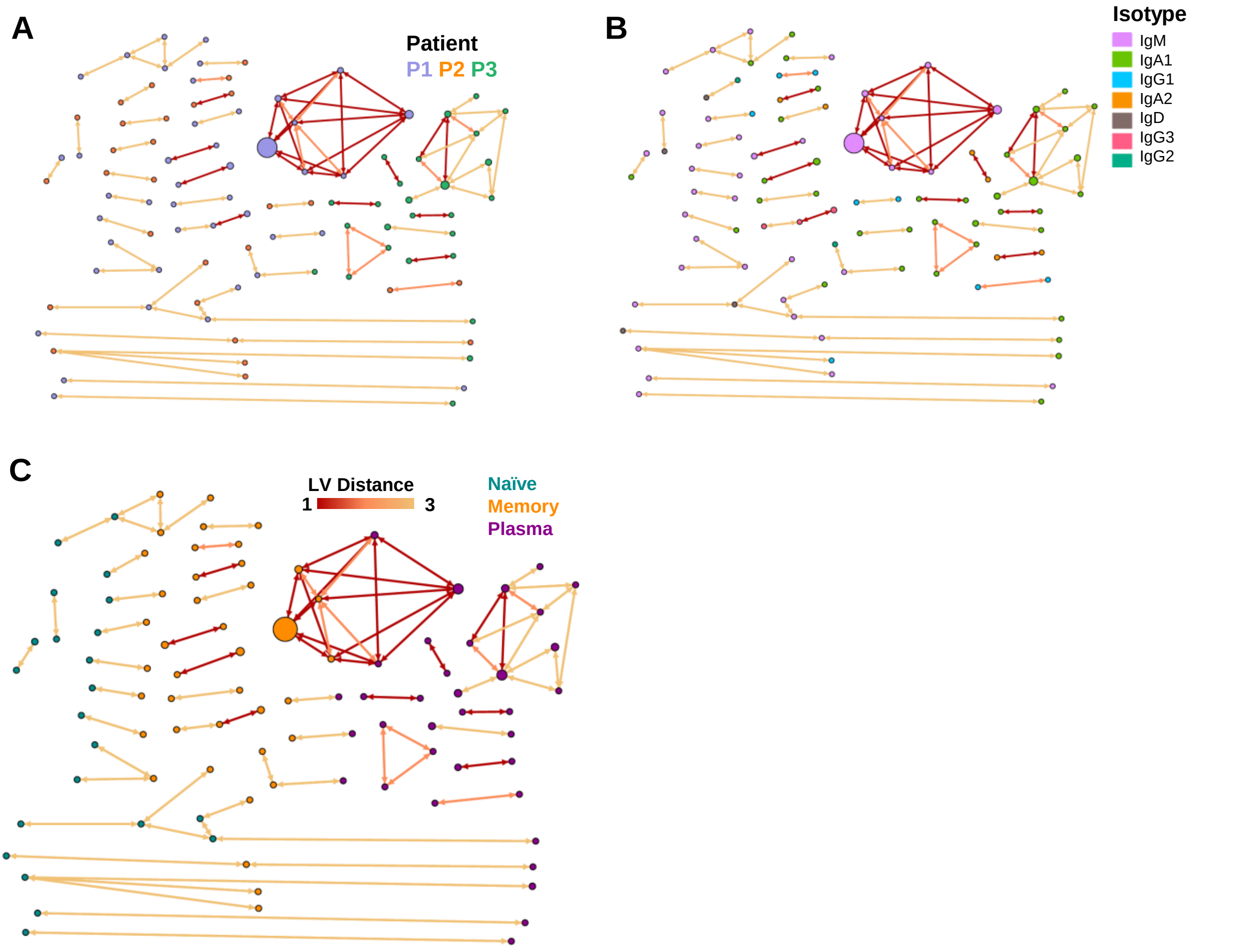

Figure S4– Plasma cell differentiation in pSS SGs does not form a continuous trajectory

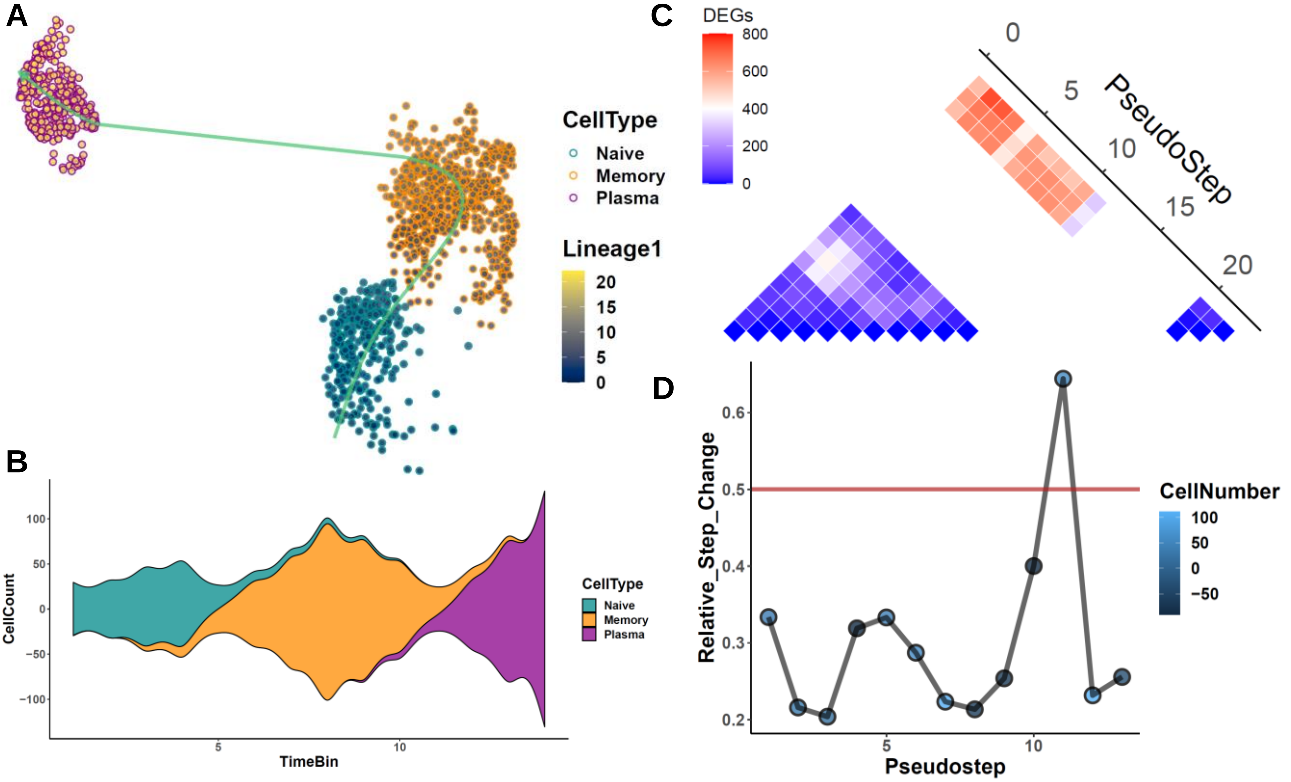

Figure S5– LASSO selection of key pathway features

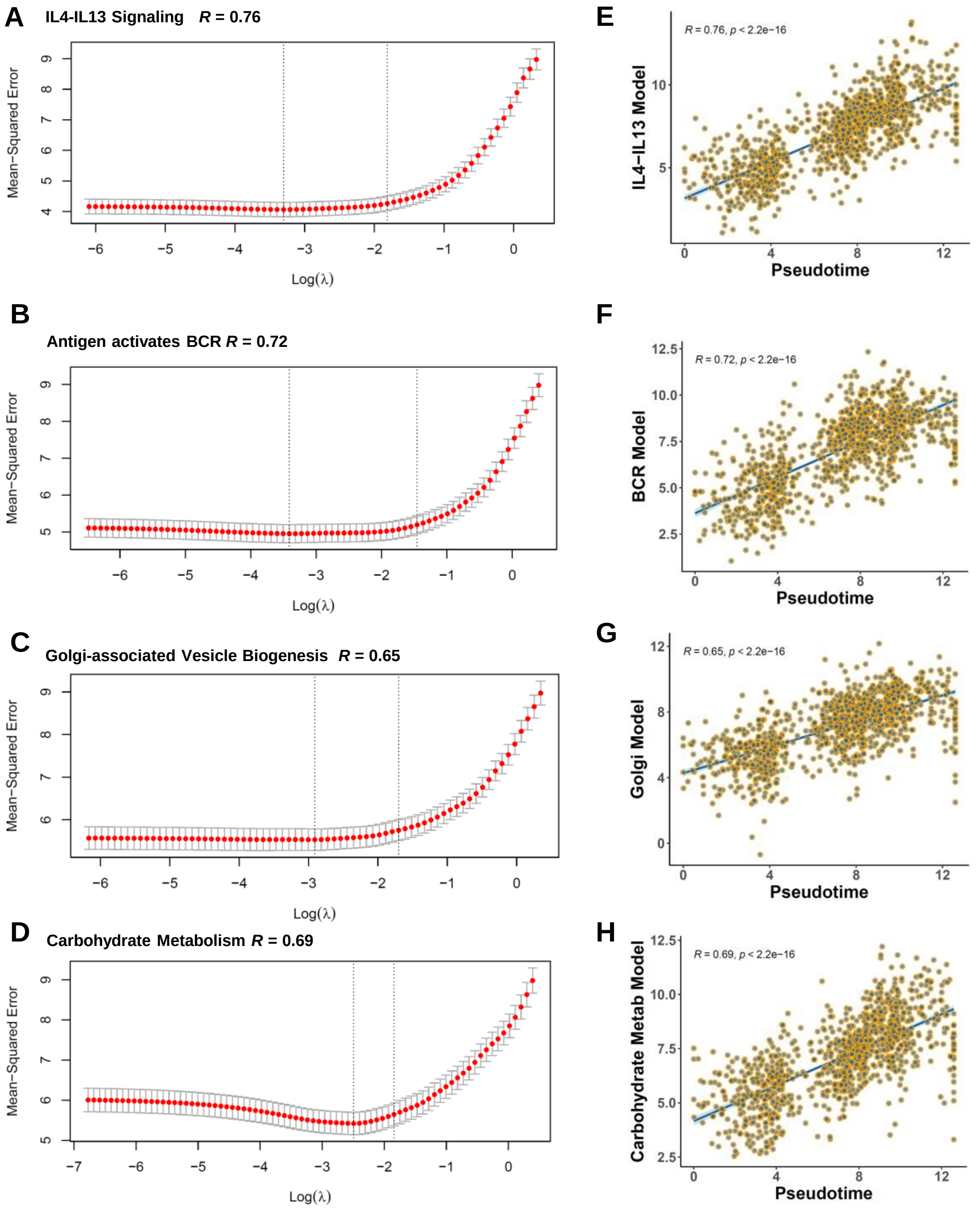

Figure S6– Gene expression change with respect to pseudotime in selected pathways

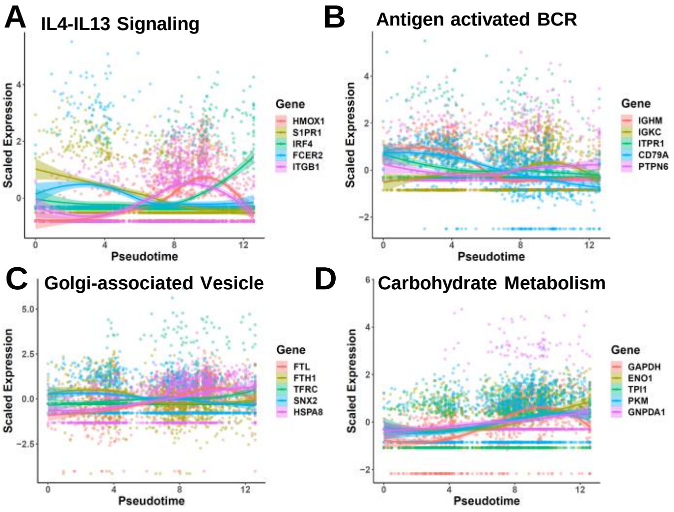

Figure S7– pSS patient glandular BCR counts is only weakly associated with disease activity metrics

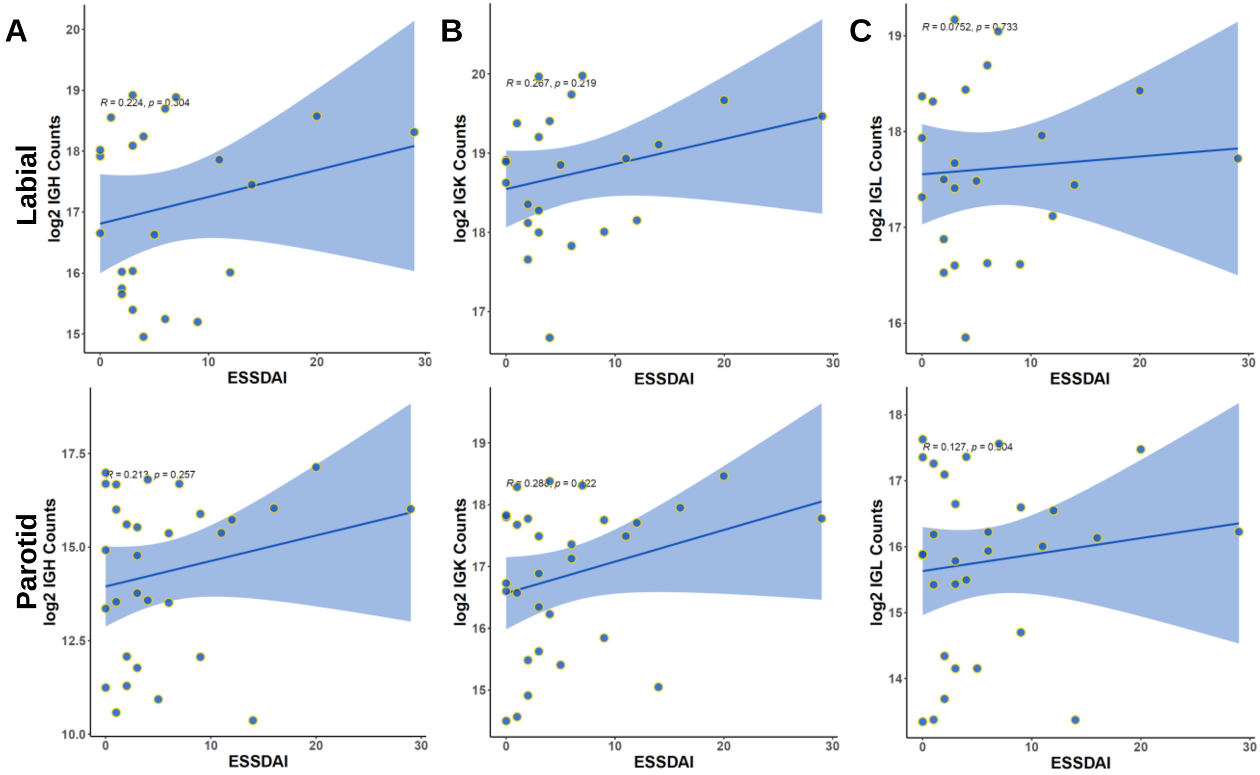

Figure S8– pSS patient BCR diversity is not clearly associated with disease activity metrics

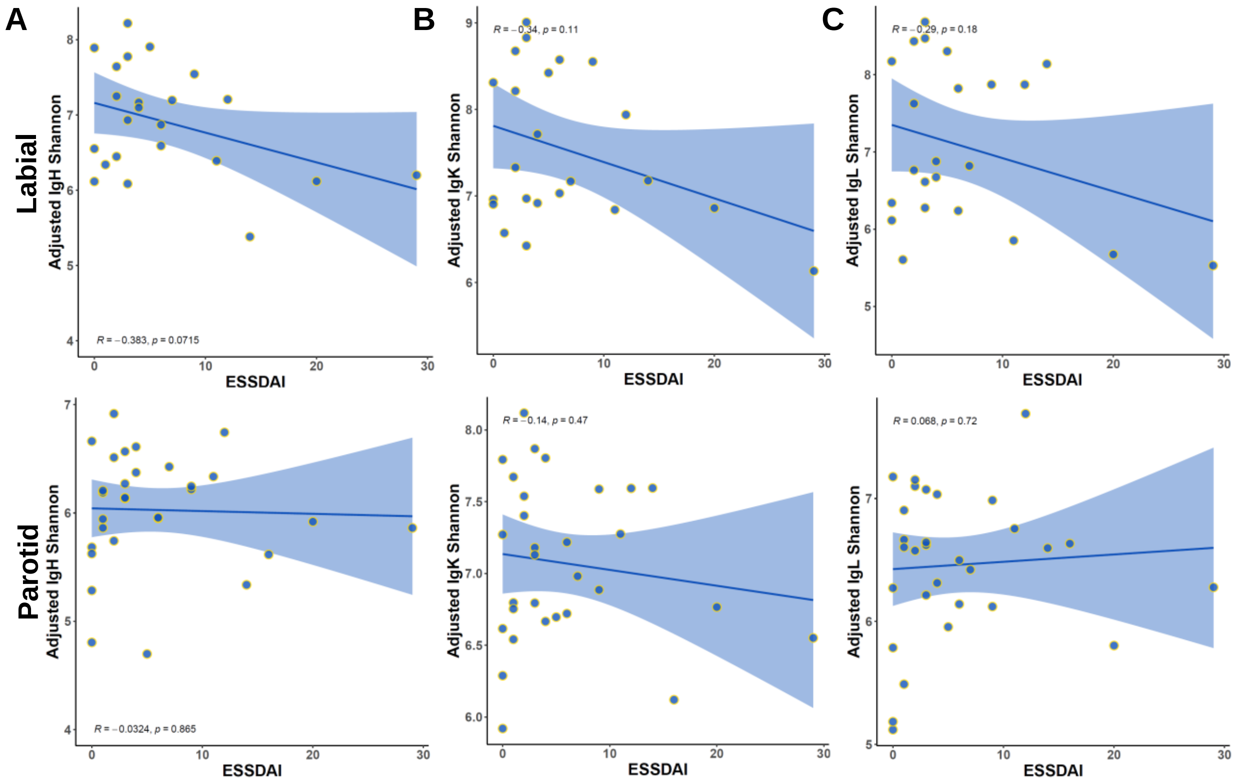

Figure S9– BCR isotype usage rates in salivary glands

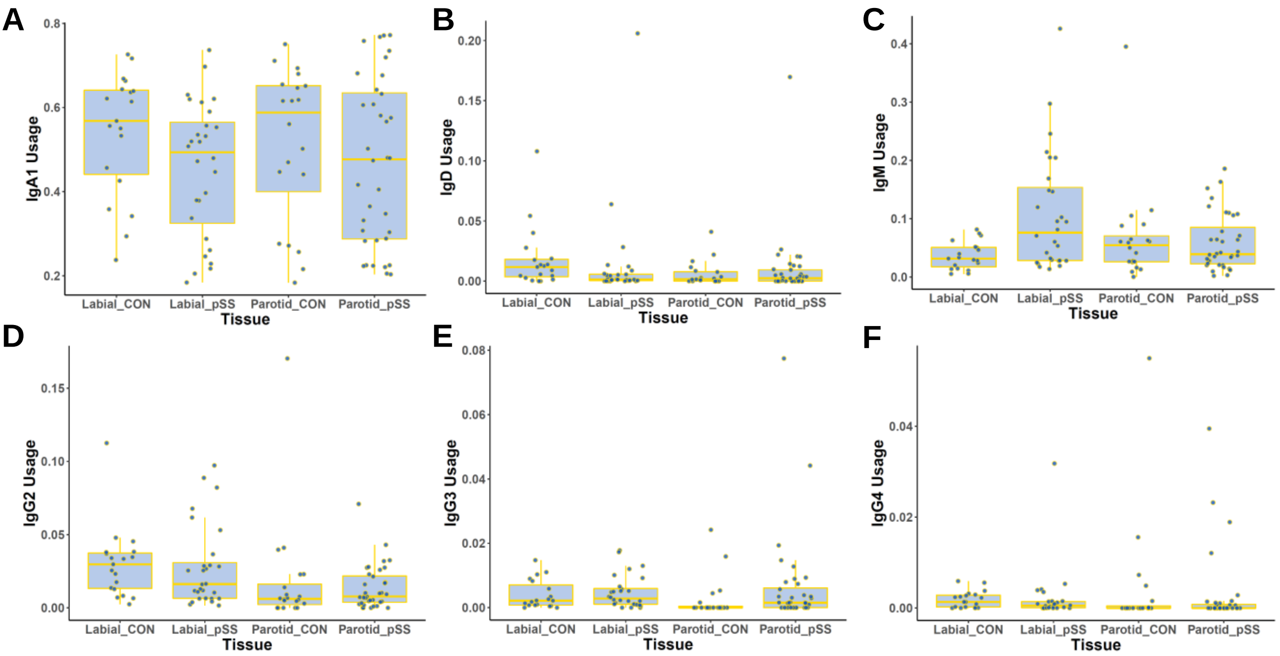

Figure S10– BCR repertoire sharing between parotid and labial glands

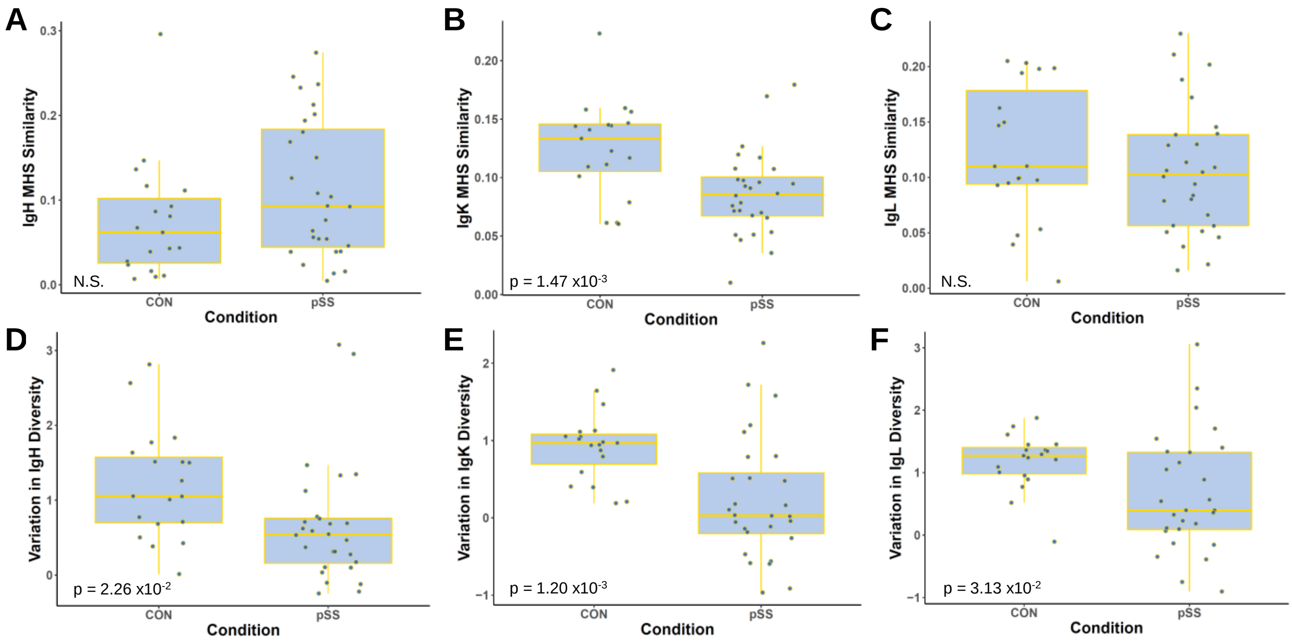

Supplement: Supplementary file 1 — Additional file 1: Figure S1. Gene expression differences between B cell populations in labial and peripheral blood samples. A) Heatmap of the top 50 differentially expressed genes marking each of the three cell types we annotated. B-D) Top differentially expressed genes among naïve B cells (B), memory B cells (C), and plasma cells (D) between peripheral blood and SG samples. DEGs calculated using Wilcoxon test, with adjusted p values < 0.05. Genes expressed in fewer than 20% of cells of at least one group were excluded. Figure S2. BCR clustering based on IgH CDR3 AA sequence. A) Network visualization as in Fig. 2A, annotated according to patient origin. B) A) Network visualization as in Fig. 2A, annotated according to heavy chain isotype usage. Figure S3. BCR clustering and associated isotypes using strict clustering of paired IgH and IgL/IgK chains. Network visualization of BCR repertoire sequences clustering by paired CDR3 amino acid sequence; sequences with Levenshtein distance between 1 and 3 are connected by edges. A) Nodes annotated according to patient origin. B) Nodes colored according to heavy chain isotype. C) Nodes colored according to cell type. Figure S4. Plasma cell differentiation in pSS SGs does not form a continuous trajectory. A) Trajectory analysis of all labial gland B cells using slingshot infers a lineage connecting naïve, memory, and plasma states. B) Stream plot of the distribution of each cell type in each bin of the pseudotime trajectory. C) Pyramid heatmap of the number of differentially expressed genes from bin-to-bin along the pseudotime trajectory. A large number of DEGs can be seen in the step that would cross from memory cell to plasma cell, causing an inferred break in the trajectory. D) Relative step change across each pseudotime step shows a break in the trajectory corresponding to the plasma cell crossover point. Since currently available trajectory inference algorithms implicitly presume continuity, a lack of continuity in a rea [file 13075_2024_3283_MOESM1_ESM.pdf]
